# Exosomes derived from miR-92a-3p-overexpressing human mesenchymal stem cells enhance chondrogenesis and suppress cartilage degradation via targeting WNT5A

**DOI:** 10.1186/s13287-018-1004-0

**Published:** 2018-09-26

**Authors:** Guping Mao, Ziji Zhang, Shu Hu, Zhiqi Zhang, Zongkun Chang, Zhiyu Huang, Weiming Liao, Yan Kang

**Affiliations:** Department of Joint Surgery, First Affiliated Hospital of Sun Yat-sen University, #58 Zhongshan 2nd Road, Guangzhou, 510080 China

**Keywords:** Exosomes, Human mesenchymal stem cells, WNT5A, miRNA-92a-3p, Osteoarthritis, Chondrocyte

## Abstract

**Background:**

WNT5A is known to be involved in the pathogenesis of osteoarthritis. This study investigated the molecular mechanism of exosomal miR-92a-3p and WNT5A in chondrogenesis and cartilage degeneration.

**Methods:**

Exosomal miR-92a-3p expression was assessed in vitro in a human mesenchymal stem cell (MSC) model of chondrogenesis and in normal and OA primary human chondrocytes (PHCs). MSCs and PHCs were treated with exosomes derived from MSC-miR-92a-3p (MSC-miR-92a-3p-Exos) or its antisense inhibitor (MSC-anti-miR-92a-3p-Exos), respectively. Small interfering RNAs (siRNAs) and luciferase reporter assay were used to reveal the molecular role of exosomal miR-92a-3p and WNT5A in chondrogenesis. The protective effect of exosomes in vivo was measured using Safranin-O and Fast Green staining and immunohistochemical staining.

**Results:**

Exosomal miR-92a-3p expression was elevated in the MSC chondrogenic exosome, while it was significantly reduced in the OA chondrocyte-secreted exosome compared with normal cartilage. Treatment with MSC-miR-92a-3p-Exos promoted cartilage proliferation and matrix genes expression in MSCs and PHCs, respectively. In contrast, treatment with MSC-anti-miR-92a-3p-Exos repressed chondrogenic differentiation and reduced cartilage matrix synthesis by enhancing the expression of WNT5A. Luciferase reporter assay demonstrated that miR-92a-3p suppressed the activity of a reporter construct containing the 3’-UTR and inhibited WNT5A expression in both MSCs and PHCs. MSC-miR-92a-3p-Exos inhibit cartilage degradation in the OA mice model.

**Conclusions:**

Our results suggest that exosomal miR-92a-3p regulates cartilage development and homeostasis by directly targeting WNT5A. This indicates that exosomal miR-92a-3p may act as a Wnt inhibitor and exhibits potential as a disease-modifying osteoarthritis drug.

**Electronic supplementary material:**

The online version of this article (10.1186/s13287-018-1004-0) contains supplementary material, which is available to authorized users.

## Background

Osteoarthritis (OA) is strongly associated with joint degenerative diseases, and leads to chronic pain, disability, and economic burden [1]. However, the molecular mechanisms of OA have not yet been fully elucidated. OA is characterized by the loss of extracellular matrix (ECM) and cartilage destruction. It is widely accepted that the development of OA is associated with pro-inflammatory cytokines, which increases the activity of matrix metalloproteinase (MMP) and a disintegrin and metalloproteinase with thrombospondin motifs (ADAMTS) [2, 3].

Recently, increasing evidence has suggested that mesenchymal stem cells (MSCs)-derived exosomes play a vital role in modulating cell migration, proliferation, differentiation, and matrix synthesis [4–6]. In 2010, it was first reported that the exosome was the active agent secreted by MSCs in response to myocardial ischemia reperfusion (I/R) injury [7]. It has also been reported that MSC exosomes mediate cartilage repair and regeneration by enhancing proliferation, attenuating apoptosis, and modulating immune reactivity [8–10]. Exosomes are small, secreted bilipid membrane vesicles of about 50–150 nm, and are thought to act as intercellular communication vehicles. Bioactive lipids, nucleic acids (mRNAs and microRNAs), and proteins are transferred by exosome and play biological responses in cells and recipient cells [7]. Evidence has shown that exosomes have no obvious immunogenicity or tumorigenicity adverse effects [11, 12]. To our knowledge, however, the specific molecular mechanism that causes MSC exosomes to promote cartilage chondrogenesis has not been investigated.

MicroRNAs (miRNAs) are an important component of the exosome and have attracted attention for many years. They bind to the 3′-untranslated regions (3′-UTRs) of target genes and suppress target gene expression [13]. While exosomal miRNAs are widely thought to mediate intercellular communication and gene regulation [14, 15], the specific molecular mechanism of MSC-derived exosomal miRNAs responsible for chondrogenic differentiation and degeneration remain unclear.

WNT5A is considered a non-canonical Wnt protein. Increasing evidence has shown that the development of articular joints, including cartilage, bone, and joint cavities, is highly dependent on Wnt signaling [16–20]. WNT5A has a dual function, acting in both chondrogenic differentiation and cartilage degradation [21]. In the early stage of cartilage formation, it activates the proliferation and inhibits the differentiation of chondrocytes [22–24]. In addition, WNT5A can activate matrix metalloproteinases (MMPs) and reduce cartilage formation and the synthesis of cartilage ECM in the late stage of chondrogenesis and in mature chondrocytes [21, 25]. WNT5A plays a key role in cartilage destruction and degradation in the pathogenesis of OA. Huang et al. showed that WNT5A can promote chondrocyte catabolic activity via non-canonical Wnt signaling in human OA cartilage [25]. Moreover, Ge et al. showed that WNT5A plays an essential role in interleukin (IL)-1β-mediated cartilage destruction via the NF-κB pathway [26, 27], and Shi et al. demonstrated that the silencing of WNT5A prevents interleukin (IL)-1β-induced collagen type II degradation in rat chondrocytes [28].

In order to elucidate the specific MSC-derived exosomal miRNAs responsible for MSC-mediated chondrogenic differentiation, in the present study, we used a miRNA microarray to determine miRNA expression profiles in exosomes during the chondrogenic differentiation of human MSCs. We observed significant upregulation of the exosomal miR-92a-3p during MSC-induced chondrogenesis. Previous studies we have demonstrated miR-92a-3p plays a key role in chondrogenesis and cartilage degradation via directly targeting noggin3,HDAC2, ADAMTS4, and ADAMTS5 [29–31]. Given the potential role of MSC-derived exosomal miRNAs in regulating cartilage homeostasis and cartilage development, we hypothesized that exosomal miR-92a-3p may play a role in both chondrogenic differentiation and OA pathogenesis. Thus, our study further aimed to determine whether MSC-derived exosomal miR-92a-3p enhances cartilage development and prevents degradation by targeting *WNT5A*.

## Methods

### Human mesenchymal stem cell isolation, culture, and induction of chondrogenesis in micromass culture

Bone marrow samples were obtained from the First Affiliated Hospital of Sun Yat-Sen University. MSCs were isolated as described previously [32]. Bone marrow samples were obtained by iliac crest aspiration from six normal human donors (mean age: 35 years; range: 32–38 years; male: 3, female: 3). Human MSCs were cultured in alpha-modified Eagle’s medium (α-MEM) (Gibco, Grand Island, NY, USA) supplemented with 10% fetal bovine serum (FBS; Gibco), 100 IU/mL penicillin, and 100 μg/mL streptomycin. Cells were cultured at 37 °C in a 5% CO2 atmosphere. Culture media were changed every 3 days. When cultures neared 80% confluence, cells were detached by treatment with 0.05% trypsin/ethylenediaminetetraacetic acid (EDTA) and passaged in culture. All hMSCs were used at passage 3 to induce hMSC chondrogenesis by micromass culture, as previously described [16]. Briefly, hMSCs were resuspended at 2 × 107 cells/mL in incomplete chondrogenic medium [97 mL human mesenchymal stem cell chondrogenic differentiation basal medium, 10 μL dexamethasone, 300 μL ascorbate, 1 mL of ITS (insulin, transferrin, selenium) supplement, 100 μL sodium pyruvate, and 100 μL proline; Cyagen Biosciences, Guangzhou, China]. Droplets of resuspended cells (12.5 μL) were carefully transferred to individual wells of a 24-well plate and incubated at 37 °C for 90 min to stimulate the adherence of cells to the plate. Droplets were divided into two groups: the first group was cultured in 500 μL incomplete chondrogenic medium per well; the second group was cultured in 500 μL complete chondrogenic induction medium, prepared by the addition of 10 μL transforming growth factor (TGF)-β 3 to 1 mL incomplete chondrogenic medium (Cyagen Biosciences). Samples were collected for experiments at selected time points.

### Characterization of MSCs and flow cytometry analysis

To identify the multiple differentiation potential of MSCs, cells were induced to differentiate in osteogenic, adipogenic, or chondrogenic differentiation medium. We used Alizarin Red, Oil Red O, and Alcian Blue staining to identify the three cell types, respectively. Flow cytometry was used to identify the surface antigens of hMSCs. CD11b, CD19, CD34, CD45, CD73, CD90, CD105, and HLA-DR (eBioscience, Inc., San Diego, CA, USA) monoclonal antibodies were used for detection. Mouse IgG monoclonal antibody was used as a negative control.

### Isolation and identification of exosomes

Exosome isolation was carried out using ultracentrifugation [33]. In brief, MSC culture supernatants were subjected to successive centrifugations at 3000 × *g* (30 min) and 10,000 × *g* (30 min). Exosomes were then pelleted at 64,000 × *g* for 110 min using an SW28 rotor (Beckman Coulter, Brea, CA, USA). Exosome pellets were resuspended in 0.32 M sucrose and centrifuged at 100,000 × *g* for 1 h (SW60Ti rotor, Beckman Coulter). The exosome pellet was then resuspended in phosphate-buffered saline (PBS). Nanosight 2000 analysis and transmission electron microscopy (TEM) were used to identify exosomes. RNA and proteins were extracted from exosomes using a Total Exosome RNA & Protein Isolation Kit (Invitrogen, Carlsbad, CA, USA) for further analysis.

### Primary chondrocyte collection, isolation, and cell culture

Degraded joint cartilage samples were obtained from patients [*n* = 6; mean age: 60.24 years; male: 3, female: 3] with OA knee joints during total knee replacement operations. Normal cartilage samples were taken from patients (n = 6; mean age: 54.46 years; male: 3, female: 3) with no previous history of OA or rheumatoid arthritis, who underwent total hip replacement surgery because of femoral neck fractures. The cartilages were dissected away from the subchondral bone and then digested by 4 mg/mL protease and 0.25 mg/mL collagenase P as described previously [30]. Cells were cultured in DMEM/F-12 (Gibco Life Technologies, Grand Island, NY, USA) containing 5% fetal bovine serum (FBS; Gibco Life Technologies), 1% penicillin and streptomycin (Gibco Life Technologies). The chondrocytes were used our in experiments within 3–7 days and without passaging to avoid dedifferentiation.

### Chondrocyte migration and proliferation assay

A scratch wound assay was used to analyze the effect of exosomes secreted by mesenchymal stem cells (MSC-Exos) and exosomes secreted by miR-92a-3p-overexpressing mesenchymal stem cells (MSC-miR-92a-3p-Exos) on the migration of chondrocytes, as described previously [34]. The images were obtained at the same position before and after incubation. Scratched areas were measured using Image-Pro Plus 6.0 software (Media Cybernetics, Bethesda, MD, USA). The effect of MSC-Exos and MSC-miR-92a-3p-Exos on the proliferation of human chondrocytes was evaluated using the Cell Counting Kit-8 (CCK-8; Dojindo, Kyushu Island, Japan) as described previously [34]. Cell proliferation curves were constructed by measuring the amount of formazan dye generated by cellular dehydrogenase activity with a microplate reader at a wave length of 450 nm.

### RNA extraction, reverse transcription, and quantitative real-time polymerase chain reaction (qRT-PCR)

RNA extraction and reverse transcription were performed as described previously [31]. Transcript levels were normalized to that of the housekeeping gene glyceraldehyde 3-phosphate dehydrogenase (GAPDH; for mRNA) or the small U6 RNA (for miRNA). The specific primers used for these analyses are listed in Table 1. Gene expression was calculated using the 2 − ^ΔΔ^Ct method, and each experiment was performed in triplicate.

**Table 1:** Primers for quantitative real-time polymerase chain reaction (qRT-PCR)

Gene		Primer sequence(5′-3′)
hsa-COL2A1	F	GCACCTGCAGAGACCTGAAAC
hsa-COL2A1	R	GCAAGTCTCGCCAGTCTCCA
hsa-COL9A1	F	GGCAGTAGAGGAGAATTAGGACC
hsa-COL9A1	R	GTTCACCGACTACACCCCTG
hsa-COL10A1	F	CATAAAAGGCCCACTACCCAAC
hsa-COL10A1	R	ACCTTGCTCTCCTCTTACTGC
hsa-SOX9	F	GGAGATGAAATCTGTTCTGGGAATG
hsa-SOX9	R	TTGAAGGTTAACTGCTGGTGTTCTG
hsa-RUNX2	F	CACTGGCGCTGCAACAAGA
hsa-RUNX2	R	CATTCCGGAGCTCAGCAGAATAA
hsa-WNT5A	F	ATTCTTGGTGGTCGCTAGGTA
hsa-WNT5A	R	CGCCTTCTCCGATGTACTGC
hsa-COMP	F	GATCACGTTCCTGAAAAACACG
hsa-COMP	R	GCTCTCCGTCTGGATGCAG
hsa-Aggrecan	F	GATGTTCCCTGCAATTACCACCTC
hsa-Aggrecan	R	TGATCTCATACCGGTCCTTCTTCTG
hsa-MMP-13	F	TCCTGATGTGGGTGAATACAATG
hsa-MMP-13	R	GCCATCGTGAAGTCTGGTAAAAT
hsa-GAPDH	F	GCACCGTCAAGGCTGAGAAC
hsa-GAPDH	R	TGGTGAAGACGCCAGTGGA
hsa/mmu-U6	F	CTCGCTTCGGCAGCACA
hsa/mmu-U6	R	AACGCTTCACGAATTTGCGT
hsa-miR-92a-3p	F	CACTTGTCCCGGCCTGTAAA
Mmu-miR-92a-3p	F	TATTGCACTTGTCCCGGCCTG
Mmu-WNT5A	F	ATGCAGTACATTGGAGAAGGTG
Mmu-WNT5A	R	CGTCTCTCGGCTGCCTATTT
Mmu-MMP13	F	ATGCATTCAGCTATCCTGGCCA
Mmu-MMP13	R	AAGATTGCATTTCTCGGAGCCTG
Mmu-COL2A1	F	CCCGCCTTCCCATTATTGAC
Mmu-COL2A1	R	GGGAGGACGGTTGGGTATCA
Mmu-Aggrecan	F	ATTTCCACACGCTACACCCTG
Mmu-Aggrecan	R	TGGATGGGGTATCTGACTGTC
Mmu-GAPDH	F	TGTGTCCGTCGTGGATCTGA
Mmu-GAPDH	R	TTGCTGTTGAAGTCGCAGGAG

### Transfection

The MSCs were transfected with miR-92a-3p mimic or inhibitor (RiboBio, Guangzhou, China) at a concentration of 50 nM; they were also transfected with WNT5A siRNA or NC (RiboBio). Lipofectamine^®^ 2000 Transfection Reagent (Gibco Life Technologies) was used to transfect cells according to the manufacturer’s instructions. Cells were then harvested after 48 h for quantitative real-time reverse transcription-polymerase chain reaction (qRT-PCR), or after 72 h for western blot analysis.

### Western blot analysis

Western blot analysis was carried out as described previously [31]. Membranes were incubated with primary antibodies against WNT5A and RUNX2 (1:1000 dilution, Cell Signaling Technology); GAPDH (1:3000, Cell Signaling Technology); aggrecan, COL2A1, and MMP13 (1:1000, Abcam, Cambridge, MA, USA); and SOX9 (1:2000, EMD Millipore, Burlington, MA, USA). The blots were then incubated with appropriate secondary antibodies (1:3000 dilution, Cell Signaling Technology) at 4 °C overnight, after which they were developed with an ECL chemiluminescence Kit (EMD Millipore).

### Immunohistochemical analysis, histology staining, and in situ hybridization

Immunohistochemical analysis was performed as described previously [31]. Briefly, samples were fixed in 4% paraformaldehyde (Sigma-Aldrich, St. Louis, MO, USA), decalcified, embedded in paraffin, and cut into 5-μm sections that were deparaffinized, rehydrated, and then stained with Safranin O/Fast Green. COL2A1, aggrecan, MMP13, and WNT5A expression was analyzed by immunohistochemistry. For in situ hybridization of miR-92a-3p expression, tissues were subsequently dehydrated with a graded series of ethanol, embedded in paraffin, and cut into 5-μm-thick sections. Sections were subjected to in situ hybridization analysis using miR-92a-3p-specific probe (Exiqon, Copenhagen, Denmark), as described in our previous study [31].

### Luciferase constructs and reporter assay

The DNA sequence of the *WNT5A* 3′-UTR was amplified by PCR using the following primers: forward, 5′-ATAGGCCGGCATAGACGCGTTAGGCAGGTTGGCTTTCATATC-3′, and reverse, 5′-AAAGATCCTTTATTAAGCTTGTGTGAACAGGGAAATTAGATC-3′. The seed sequences were mutated using standard PCR techniques with the following primers: forward, 5′-GTATTC**CGATGTA**AAAACACAATGAACCTTTAGTTTC-3′, and reverse, 5′-GTGTTTT**TACATCG**GAATACAAGTTATTGTGCTTTTCAAA-3′. The amplified DNA sequences were inserted into the pmiR-RB-REPORT Vector (OBIO, Shanghai, China) to generate wild-type or mutant *WNT5A* 3′-UTR luciferase vectors. For the dual luciferase assay, 1.2 × 10^4^ HEK293 cells in a 96-well plate were transfected with 50 nM miR-92a-3p or miR-NC (RiboBio). The cells were then co-transfected with 2 μg/mL of the wild-type or mutant *WNT5A* 3′-UTR vector. Luciferase activity was measured 48 h post-transfection using the Dual-Luciferase Reporter Assay System (Promega, Madison, WI, USA). Firefly luciferase activity was then normalized to the corresponding *Renilla* luciferase activity. Luciferase assays were performed in quadruplicate and repeated in three independent experiments.

### Collagenase-induced OA model

All procedures were approved by the Animal Research Committee of the First Affiliated Hospital of Sun Yat-sen University. Six-week-old female C57B/L10 mice were randomized into four groups: normal group (*n* = 10), MSC-Exos group (n = 10), MSC-miR-92a-3p-Exos group (n = 10), and OA group (n = 10). The following operations must be performed in a sterile environment to avoid joint infection. On day 0, the mice of the MSC-Exos, MSC-miR-92a-3p-Exos, and OA groups were induced with the OA model with collagenase VII (12 U of collagenase VII in 8 μl saline, Clostridium histolyticum; Sigma-Aldrich) as described previously [34]. Mice in the normal groups were injected with 8 μl saline as control. On days 7, 14, and 21, the MSC-Exos and MSC-miR-92a-3p-Exos groups were injected with 15 μl MSC-Exos (500 μg/mL) or 15 μl MSC-miR-92a-3p-Exos in PBS (500 μg/mL). Mice in the OA and normal groups were injected with 15 μl PBS at each time point. On day 28, mice were euthanatized for further analysis.

### Statistical analysis

All experiments were performed with at least three biological replicates. Data were expressed as the mean ± standard deviations (SD). Both parametric and nonparametric inferential statistics were utilized in this study depending on whether the data was normally distributed. The independent *t* test and Mann-Whitney *U* test were used to identify differences between groups as appropriate. One-way analysis of variance (ANOVA) and Kruskal-Wallis tests were carried out for multiple group comparisons. Data analyses were performed using SPSS Version 20 (IBM Corporation, Armonk, NY, USA). Statistical significance was determined at level of *P < 0.05.*

## Results

### Identification of MSCs and MSC-Exos

**Fig. 1:**
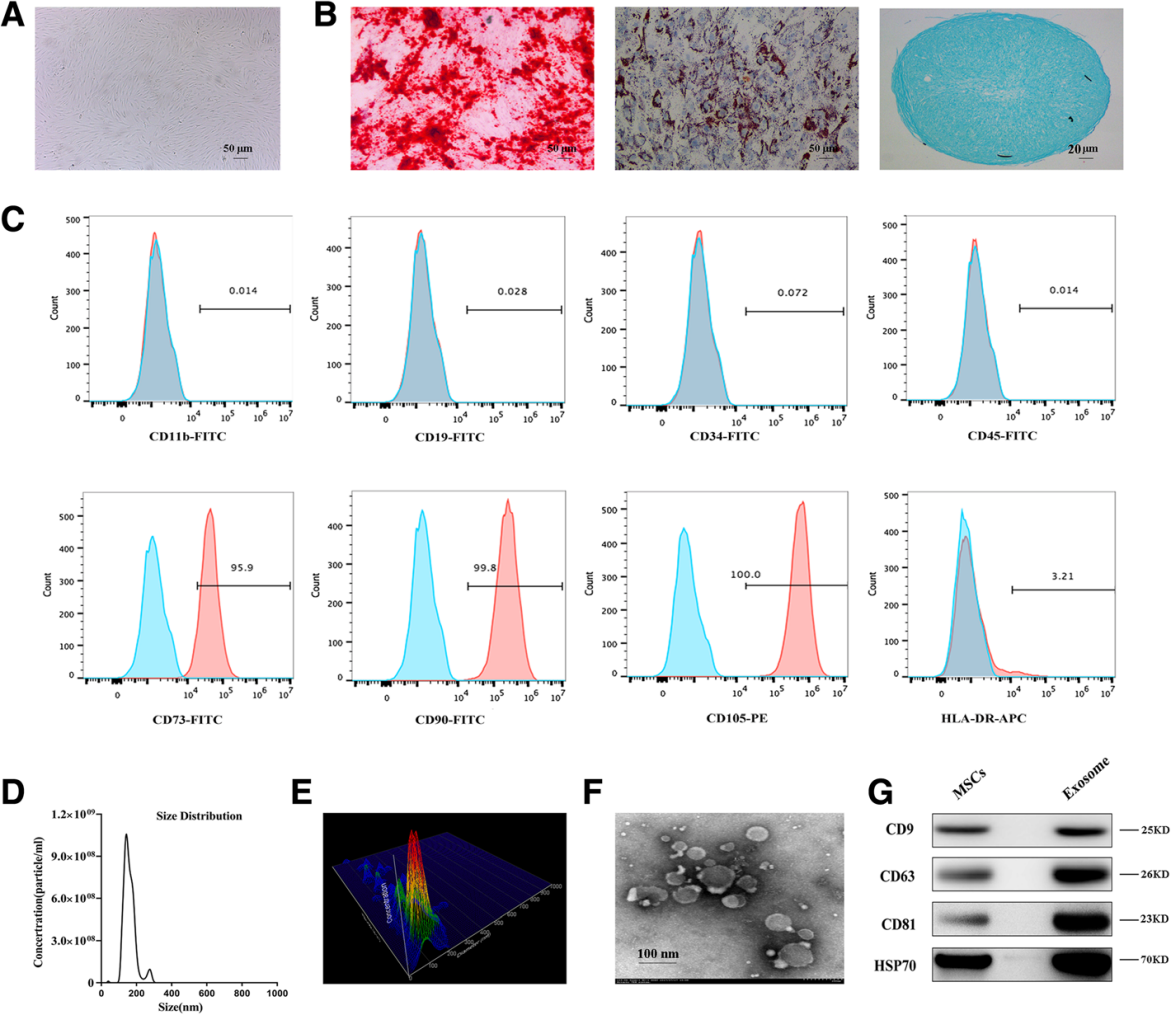
Characterization of human mesenchymal stem cells (MSCs) and exosomes. **a** MSCs exhibited a representative spindle-like morphology. **b** MSCs exhibited multidifferentiation capacity for osteogenesis, adipogenesis, and chondrogenesis. **c** Flow cytometric analysis of mesenchymal positive markers, such as CD73, CD90, and CD105, and negative markers, such as CD11b, CD19, CD34, CD45, and HLA-DR. *Blue histograms* represent the isotype controls, and the *red solid peak* represents the marker indicated. This experiment was repeated three times independently. **d-e** Particle size distribution of exosomes was measured by Nanosight. **f** Morphology of exosomes observed by transmission electron microscopy (TEM). **g** Exosome surface markers (CD9, CD63, CD81, and HSP70) measured using western blotting. This experiment was repeated three times independently, and representative results are shown

MSCs were extracted during iliac crest aspiration and were isolated and identified at passage 5 (P5) for use in subsequent experiments. After reaching 80–90% confluence, MSCs showed a robust proliferation capability and appeared to be a relatively homogeneous population of spindle-shaped cells (Fig. 1a). The MSCs could readily be induced to differentiate into the osteogenic, adiopogenic, and chondrogenic lineages, when cultured in their respective medium. Osteogenic potential was confirmed by the staining of calcium mineral deposits with Alizarin Red (Fig. 1b, left panel), while adipogenic potential was evaluated by the observation of small cytoplasmic lipid droplets stained with Oil Red O (Fig. 1b, middle panel). Chondrogenic potential was confirmed by sectioning beads and staining sulfated glycosaminoglycans using Alcian Blue (Fig. 1b, right panel). Following the criteria for identifying stem cells, we analyzed the surface markers of the MSCs. Analysis of surface antigen expression using flow cytometry demonstrated that the MSCs were positive for CD73, CD90, and CD105 and negative for CD11b, CD19, CD34, CD45, and HLA-DR (Fig. 1c). Nanosight analysis showed that the size of the majority of MSC-Exos was approximately 50–150 nm (Fig. 1d, e). TEM clearly revealed that MSC-Exos exhibited a cup-shaped or round morphology with a diameter of 50–150 nm (Fig. 1f). Western blotting analyses indicated that the MSC-Exos expressed exosomal markers such as CD9, CD63, CD81, and HSP70 (Fig. 1g).

### MiRNA expression profiles in exosomes before and after chondrogenic differentiation of MSCs

**Fig. 2:**
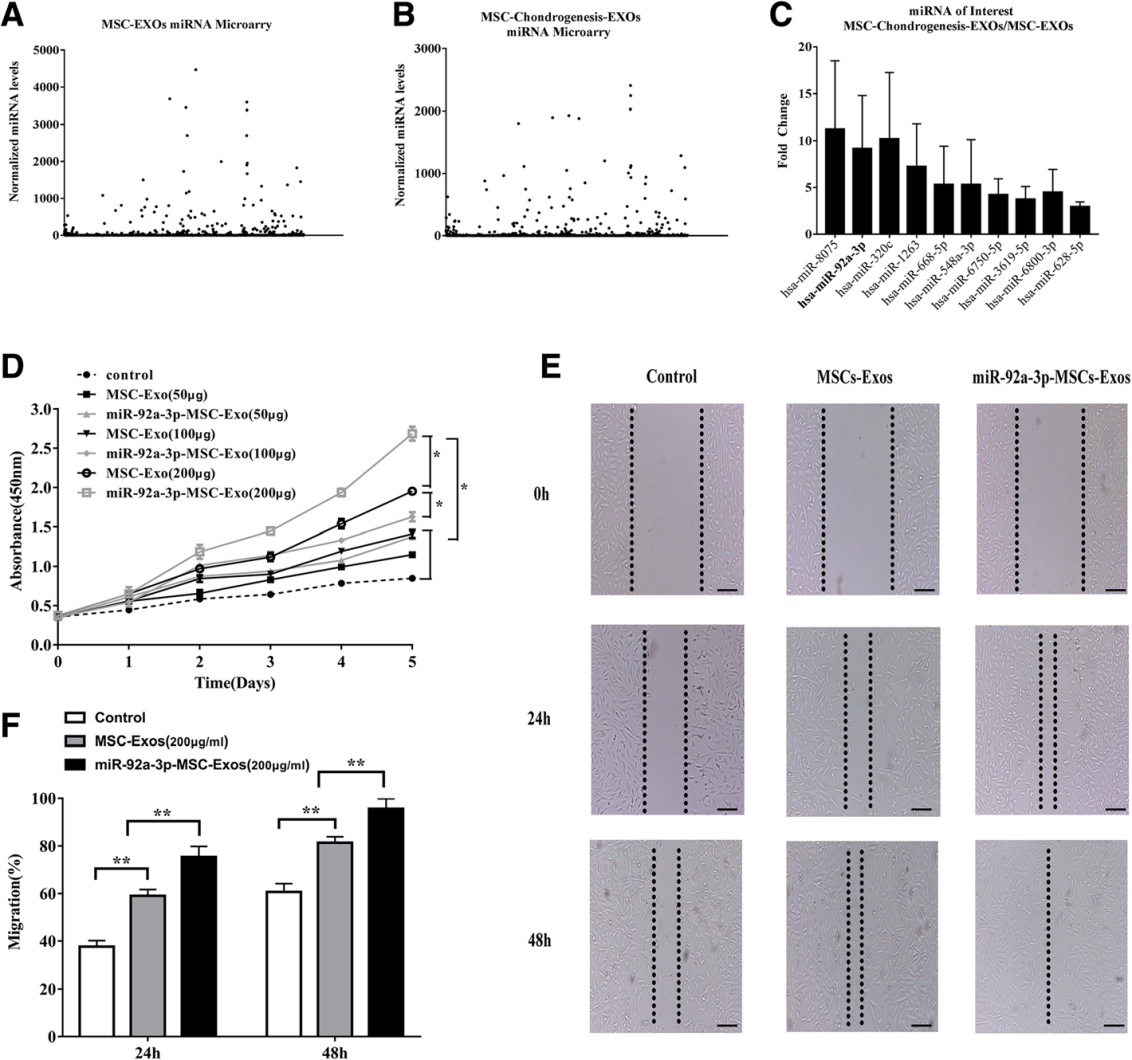
Effects of miR-92a-3p-MSC-Exos on migration and proliferation in chondrocytes. **a–b** Normalized gene expression levels in MSC-Exos and chondro-differentiated MSC-Exos detected by miRNA microarray. Three independent samples were used. **c** Gene expression levels of highly expressed genes in MSC-chondro-differentiated-Exo vs MSC-Exos and gene expression levels of miR-92a-3p. **d** MSC-Exos and miR-92a-3p-MSC-Exos were treated with chondrocytes at different doses. Quantitative data are presented as means ± standard deviations of three independent experiments. *P < 0.05. **e** Light microscopy images of scratch wound assays. **f** Quantitative analysis of migration rates at 24 and 48 h. Quantitative data are presented as means ± standard deviations of three independent experiments. **P < 0.01

The expression profiles of miRNAs in exosomes before and after chondrogenic differentiation were detected using a miRNA microarray. SAM statistical software was used to identify differentially expressed miRNAs of exosomes between undifferentiated MSCs and chondrogenically differentiated MSCs. The differentially expressed miRNAs from all three paired samples are shown in Fig. 2a–b and Additional file 1. Among the miRNAs that were consistently differentially expressed in all three paired samples, 36 were upregulated (fold change > 2) and 105 were downregulated (fold change <− 2) in chondro-differentiated MSC-Exos compared to levels in MSC-Exos, as shown in Additional file 1. Among the top ten most significantly differentially expressed exosomal miRNAs (Fig. 2c), exosomal miR-92a-3p was upregulated 7.89-fold in exosomes after chondrogenic differentiation of MSCs. These data indicate that exosomal miRNAs play an important role in cartilage development.

### Chondrocyte proliferation and migration assays

Proliferation was evaluated following the stimulation of chondrocytes with various doses of MSC-Exos, MSC-miR-92a-3p-Exos, or vehicle (PBS) as a control. At a concentration of 200 μg exosomes/mL, chondrocytes cultured with either MSC-Exos or MSC-miR-92a-3p-Exos showed greater proliferation than those incubated with PBS or other doses of exosomes. Moreover, the MSC-miR-92a-3p-Exos had a more potent effect on chondrocyte proliferation than the MSC-Exos (Fig. 2d). The chondrocytes were treated with 200 μg exosomes/mL MSC-Exos or MSC-miR-92a-3p-Exos. Scratch wound assays indicated that while both MSC-Exos and MSC-miR-92a-3p-Exos significantly enhanced the motility of chondrocytes, MSC-miR-92a-3p-Exos were more effective than MSC-Exos in increasing motility at 24 and 48 h (Fig. 2e, f).

### Opposing expression patterns of exosomal miR-92a-3p and *WNT5A* during chondrogenic differentiation of MSCs

**Fig. 3:**
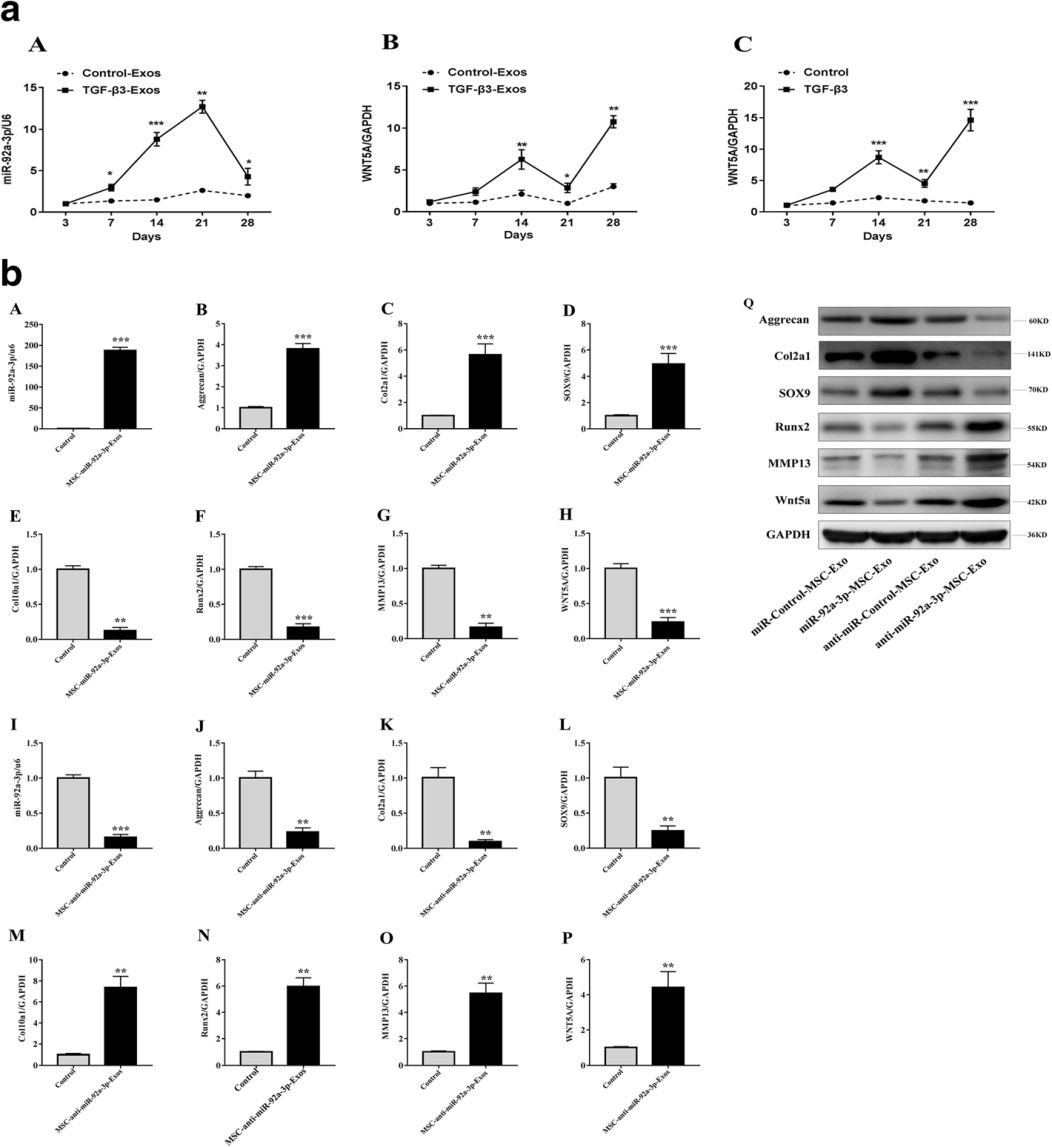
**a** Expression of miR-92a-3p and *WNT5A* mRNA in MSC -chondro-differentiated-Exos. MSCs were induced to undergo chondrogenesis with TGF-β3 for 3, 7, 14, 21, and 28 days as indicated (*solid lines*). Gene expression levels of miR-92a-3p (A) and *WNT5A* (B) in chondro-differentiated MSC-Exos and *WNT5A* (C) in chondro-differentiated MSCs were determined by qRT-PCR. MSCs cultured without TGF-β3 at corresponding time points served as negative controls (*broken lines*). Quantitative data are presented as means ± standard deviations of three independent experiments. U6 and GAPDH were used as endogenous controls. Data are presented as means ± standard deviations of six samples. *P < 0.05, **P < 0.01, ***P < 0.001*.* **b** MSC-miR-92a-3p-Exos regulate cartilage-specific gene expression during chondrogenesis. MSCs were treated with TGF-β3 to induce chondrogenesis and treated with MSC**-**miR-92a-3p-Exos or MSC-anti-miR-92a-3p-Exos. Expression levels of miR-92a-3p (A, I) were determined by qRT-PCR. Expression levels of aggrecan (B, J, Q), COL2A1 (C, K, Q), SOX9 (D, L, Q), COL10A1 (E, M), RUNX2 (F, N, Q), MMP13 (G, O, Q), and WNT5A (H, P, Q) were determined by both qRT-PCR and western blotting. U6 and GAPDH were used as endogenous controls. Data represented as means ± standard deviations of six samples. **P < 0.01, ***P < 0.001

The MSCs were induced to differentiate into chondrocytes in vitro using TGF-β3. We observed a rapid upregulation of exosomal miR-92a-3p in chondrogenic MSCs beginning on day 7, which peaked at 21 days and was followed by a marked decrease in expression at day 28 (Fig. 3a. A). Concurrently, there was a significant upregulation of *WNT5A* in exosomes and tissues on days 21–28, suggesting that miR-92a-3p may affect *WNT5A* expression (Fig. 3a. B, C).

### Exosomal miR-92a-3p regulates cartilage development in MSCs during chondrogenesis

To further investigate whether exosomal miR-92a-3p regulates chondrogenesis of MSCs, we overexpressed or inhibited miR-92a-3p in MSCs. The MSCs were transfected with MSC-miR-92a-3p-Exos or MSC-anti-miR-92a-3p-Exos. Next, MSC cultures near 90% confluence were induced to differentiate into chondrocytes and were incubated with MSC-miR-92a-3p-Exos or MSC-anti-miR-92a-3p-Exos in micromass for 14 days (Fig. 3b). The mRNA and protein expression levels of aggrecan, COL2A1, and SOX9 (Fig. 3b.B–D) were significantly upregulated and those of COL10A1, RUNX2, MMP13, and WNT5A were significantly downregulated in the MSC-miR-92a-3p-Exos treatment as assessed by qRT-PCR and western blotting (Fig. 3b.Q). In contrast, COL10A1, RUNX2, MMP13, and WNT5A levels were significantly upregulated (Fig. 3b.N-P and Fig. 3b.Q) and COL2A1, aggrecan, and SOX9 were significantly downregulated (Fig. 3b.J–L and Fig. 3b.Q) in MSC-anti-miR-92a-3p-Exos. These data indicate that miR-92a-3p may target *WNT5A* to promote SOX9 and aggrecan expression and enhance cartilage development.

### Expression levels of exosomal miR-92a-3p and WNT5A in normal and OA cartilage

**Fig. 4:**
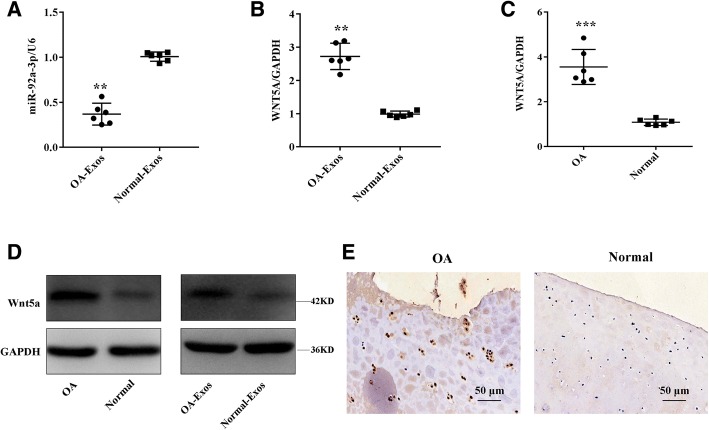
Expression of miR-92a-3p and WNT5A in normal and OA cartilage. Relative miR-92a-3p and *WNT5A* (**a**, **b**) mRNA levels in normal and OA cartilage-secreted exosomes and (**c**) *WNT5A* mRNA levels in normal and OA cartilage tissues were determined by qRT-PCR. U6 and GAPDH were used as endogenous controls. Each *dot* represents a value from a single experiment involving a single donor. The *bar* shows the mean and 95% confidence intervals of the values from six different donors per group. **P < 0.01, ***P < 0.001. WNT5A protein levels in normal cartilage and OA cartilage-secreted exosomes or tissues were determined by western blotting and immunohistochemistry using anti-WNT5A monoclonal antibody (**d**, **e**). GAPDH was used as an endogenous control. Data shown are representative of results of six normal and OA cartilage samples. Scale bar 50 μm

To determine the expression of exosomal miR-92a-3p during the progression of OA, we compared its expression levels in normal and OA cartilage-secreted exosomes. Exosomal miR-92a-3p expression was significantly reduced in OA cartilage compared with levels in normal cartilage (Fig. 4a). However, OA cartilage exhibited higher levels of *WNT5A* mRNA (Fig. 4b, c) and protein compared with levels in normal cartilage (Fig. 4d, e).

### Exosomal miR-92a-3p maintains the function of articular chondrocytes

**Fig. 5:**
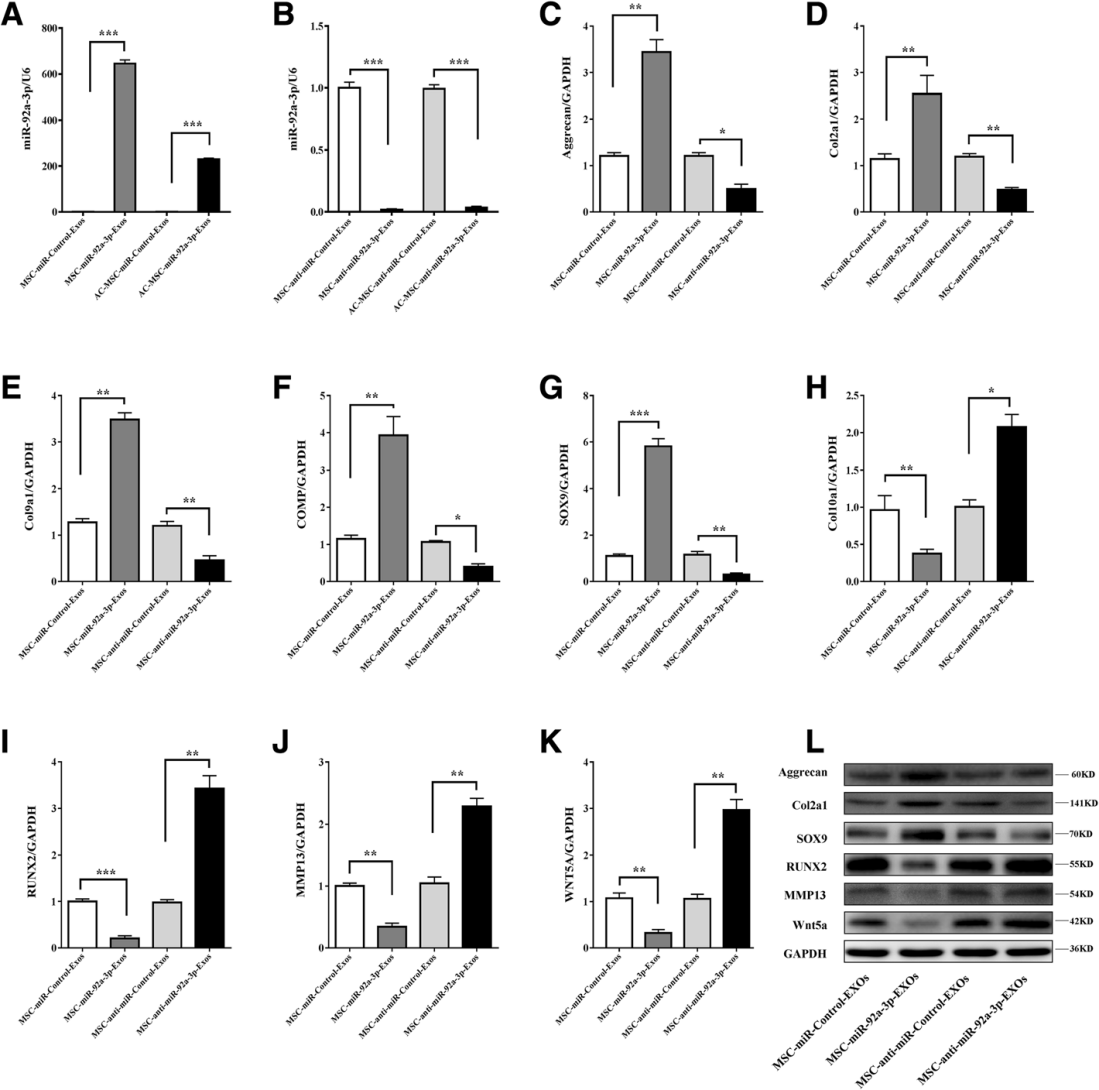
Responses of articular chondrocytes to stimulation by MSC- miR-92a-3p-Exos. MiR-92a-3p (**a**, **b**) expression level changes in exosomes after treatment of chondrocytes with MSC-miR-Control-Exos, MSC-miR-92a-3p-Exos, MSC-anti-miR-Control-Exos or MSC-anti-miR-92a-3p-Exos were determined by qRT-PCR. U6 was used as an endogenous control. Gene expression levels of aggrecan (**c**), COL2A1 (**d**), COL9A1 (**e**), COMP (**f**), SOX9 (**g**), COL10A1 (**h**), RUNX2(**i**), MMP13(**j**), and WNT5A(**k**) were estimated by qRT-PCR. Quantitative data are presented as means ± standard deviations of three independent experiments. *GAPDH* was used as an internal control. *P < 0.05, **P < 0.01, ***P < 0.001. **l** Protein expression levels of aggrecan, col2a1, SOX9, RUNX2, MMP13, and Wnt5a were visualized by western blotting. GAPDH was used as an endogenous control

To determine the effect of exosomal miR-92a-3p on chondrocyte function, we treated chondrocytes with MSC-miR-92a-3p-Exos or MSC-anti-miR-92a-3p-Exos (200 μg exosomes/mL). We found that MSC-miR-92a-3p-Exos significantly upregulated the mRNA and protein expression levels of aggrecan, COL2A1, COL9A1, COMP, and SOX9 and decreased the expression levels of COL10A1, RUNX2, MMP13, and WNT5A (Fig. 5). In contrast, MSC-anti-miR-92a-3p-Exos accelerated cartilage matrix degradation and increased WNT5A expression. These data indicate that MSC-miR-92a-3p-Exos may slow the progression of OA and maintain cartilage stability.

### SiWNT5A promotes cartilage-specific gene expression

**Fig. 6:**
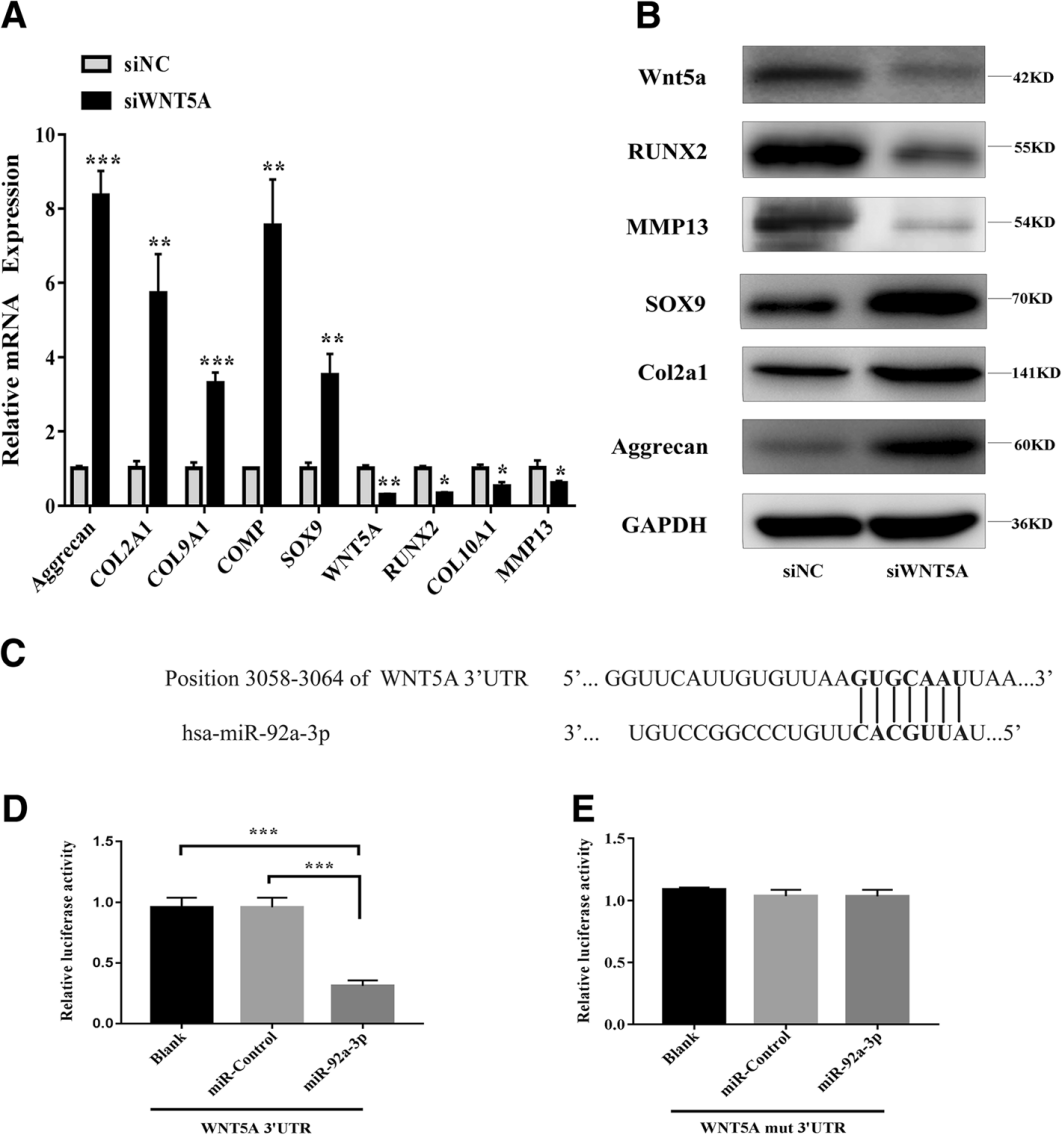
PHCs were transfected with siWNT5A. **a** The gene expression levels of aggrecan, COL2A1, COMP, COL9A1, SOX9, RUNX2, COL10A1, MMP13, and WNT5A were determined by qRT-PCR. Quantitative data are presented as means ± standard deviations of three independent experiments. GAPDH was used as an internal control. *P < 0.05, **P < 0.01, ***P < 0.001. **b** Expression of WNT5A, RUNX2, MMP13, SOX9, COL2A1, and aggrecan as visualized by western blot. GAPDH was used as an internal control. **c** Alignment of miR-92a-3p and the 3′-UTR of *WNT5A,* a potential miR-92a-3p target. **d**, **e** A luciferase reporter carrying the 3’-UTR of wild-type (Luc-WNT5A-UTR) or mutant (Luc-WNT5A-UTR-mut) *WNT5A* was introduced into 293 T cells along with negative miR-control (NC) or miR-92a-3p. Cells were harvested 48 h later for luciferase assays. Quantitative data are presented as means ± standard deviations of three independent experiments. ***P < 0.001

To perform the converse of the above experiments, a siRNA approach was used to reduce the levels of WNT5A. Chondrocytes were transfected with siWNT5A, which resulted in the downregulation of *WNT5A* mRNA levels, as shown by qRT-PCR (Fig. 6a). This reduction was well correlated with a decrease in cartilage WNT5A protein expression, as well as increases in the expression of COL2A1, aggrecan, and SOX9, as shown in Fig. 6b. These data indicate that the downregulation of WNT5A promotes increases in the expression of cartilage-specific proteins.

### MiR-92a-3p directly targets the 3′-UTR of *WNT5A* mRNA

To further clarify the molecular mechanisms that underlie the regulation of *WNT5A* expression by miR-92a-3p, we analyzed the sequence of the 3′-UTR of the human *WNT5A* gene. Bioinformatics software such as Targetscan (http://www.targetscan.org) and miRanda (http://www.microrna.org) revealed that the 3′-UTR of human *WNT5A* contains a potential miR-92a-3p binding site (Fig. 6c). Luciferase reporter assays with the wild-type or mutant 3′-UTR of *WNT5A* were performed in the presence or absence of miR-92a-3p overexpression. Transfection with miR-92a-3p resulted in reduced luciferase activity (indicating reduced transcription of *WNT5A*) by binding to the wild-type 3′-UTR, while the mutant 3′-UTR sequence prevented the binding of miR-92a-3p. This suggests that *WNT5A* is a target of miR-92a-3p-mediated repression (Fig. 6d, e).

### MSC-miR-92a-3p-Exos inhibits cartilage degradation

**Fig. 7:**
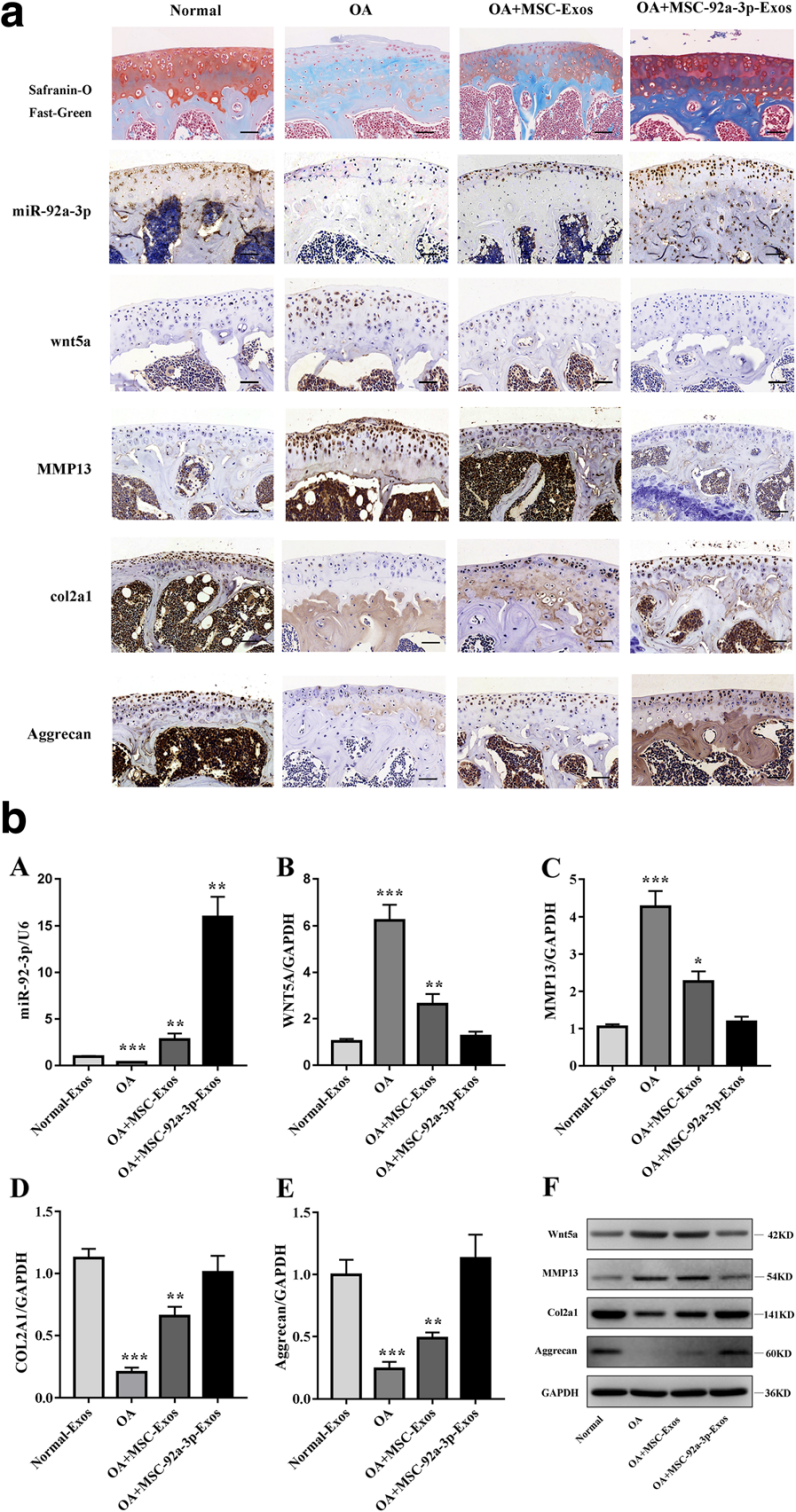
MSC-miR-92a-3p-Exos inhibits cartilage degradation. **a**. Sections of femoral condyle (*n* = 10 per group) were stained using Safranin-O and Fast Green and were stained the miR-92a-3p probe for analysis of miR-92a-3p expression. Femoral condyle sections (*n* = 10 per group) stained with col2a1, aggrecan, wnt5a, or MMP13 as primary antibodies (scale bar: 50 μm). **b**. Gene expression levels of miR-92a-3p (A), WNT5A (B), MMP13 (C), COL2A1 (D), and aggrecan (E) were estimated by qRT-PCR. Quantitative data are presented as means ± standard deviations of three independent experiments. *GAPDH* was used as an internal control. *P < 0.05, **P < 0.01, ***P < 0.001. (F) Protein expression levels of aggrecan, col2a1, MMP13, and Wnt5a were visualized by western blotting. GAPDH was used as an endogenous control

To investigate the role of exosomal miR-92a-3p in osteoarthritis in vivo, we built the OA mice model (Fig. 7). In the OA group, the cartilage matrix consisting of col2a1 and aggrecan in cartilage were obviously decreased, while expression of wnt5a and MMP13 were upregulated on the cartilage surface. In the OA + MSC-Exos group, cartilage matrix loss was much less severe compared to the OA group. In the OA + MSC-miR-92a-3p-Exos group, cartilage matrix was still present but the severity was very mild. The col2a1 and aggrecan were slightly less than in the normal groups but significantly better than in the OA group or the OA + MSC-Exos group. These histological staining results are consistent with WNT5A, MMP13, COL2A1, and aggrecan mRNA and protein expression. These data indicated that MSC-miR-92a-3p-Exos inhibit the progression of early OA and prevented the severe damage to knee articular cartilage in the OA mice model.

## Discussion

Osteoarthritis (OA) is a common, debilitating disease that is associated with a large social and economic burden. Disease progression is usually slow but can ultimately lead to joint failure with pain and disability [35, 36]. Current therapies only relieve the symptoms, but cannot stop or reverse cartilage degeneration and destruction [37, 38] . Previous studies showed that MSC exosomes, which contain miRNAs, mRNAs, and proteins, are likely to be important mediators in cell–cell communication to induce changes in cell functions and processes [39, 40]. Exosomal miRNAs are important components of MSC exosomes, and modulate a variety of injuries and diseases, including OA. For example, some of the miRNAs found in MSC exosomes, such as miR-23b and miR-320may be therapeutic for their roles in modulating matrix synthesis [41, 42].

In this study, we verified the presence of miRNAs in exosomes isolated from the supernatant of MSC culture. We found that miR-8075, miR-92a-3p, miR-320c, miR-1263, miR-668-5p, miR-548a-3p, miR-6750-5p, miR-3619-5p, and miR-628-5p were significantly upregulated in three paired exosomal samples from MSCs induced to undergo chondrogenesis. In contrast, miR-1225-5p, miR-7150, miR-466, miR-7107-5p, and miR-329-3p were significantly downregulated in these exosomes. Furthermore, we are the first to discover that exosomal miR-92a-3p mediates cartilage development and degradation via WNT5A. Our data showed that exosomal miR-92a-3p was expressed in vitro, particularly during the late stage of chondrogenic differentiation. Previous studies have shown that miR-92a-3p enhances SOX9 and COL2A1 expression, directly targets *HDAC2*, and delays the progression of OA by suppressing ADAMTS4 and ADAMTS5 [30, 31].

In further experiments, we found that treatment with MSC-miR-92a-3p-Exos promoted cartilage development when MSCs were induced to differentiate into chondrocytes. Furthermore, *WNT5A* was validated as a target of miR-92a-3p through bioinformatics analysis, western blot assays, and luciferase reporter assays. Moreover, treatment with MSC-anti-miR-92a-3p-Exos enhanced WNT5A expression in PHCs and was associated with reduced collagen deposition and COL2A1 expression, while treatment with MSC-miR-92a-3p-Exos markedly suppressed WNT5A production and was associated with increased collagen deposition and COL2A1 expression. Therefore, these data suggest that exosomal miR-92a-3p plays an active role in the regulation of WNT5A during chondrogenesis and degeneration.

Wnt proteins affect cellular homeostasis by regulating cell proliferation, cell fate determination and differentiation [43]. WNT5A plays dual roles in matrix homeostasis, including catabolic role and anabolic effect [27, 44, 45]. For example, it has been demonstrated that Wnt5a could induce MMPs expression and inhibit collagen II expression in chondrocytes [27, 44]. However, Kumawat et al. reported that Wnt5a signaling is activated by TGF-β and necessary for TGF-β-induced ECM production in smooth muscle cells [45]. Tao et al. also demonstrated Wnt5a and Wnt5b carried by exosomes activated YAP via the alternative Wnt signaling pathway and enhanced proliferation and migration of chondrocytes with the side-effect of significantly decreasing ECM secretion [15]. In our study, we demonstrated the expression profile of WNT5A during MSC chondrogenesis and found that WNT5A expression was maintained at a stable level in the early stage (days 3–14) of chondrogenic differentiation, which may enhance chondrogenesis. However, expression of WNT5A rapidly increased in the late stage (days 14–21), which may promote MMP13 expression, leading to the loss of aggrecan, COL2A1, COL9A1, and COMP expression. Thus, our data are consistent with previous reports of the dual nature of WNT5A function during cartilage development and in disease. Moreover, PHCs were treated with MSC-miR-92a-3p-Exos or MSC-anti-miR-92a-3p-Exos and found that MSC-miR-92a-3p-Exos promote the expression of cartilage matrix proteins, such as aggrecan, COL2A1, and COMP, and suppress the expression of MMP13 and WNT5A. Further, the silencing of WNT5A blocks the catalytic activity of WNT5A and suppresses cartilage degeneration [28]. The Wnt inhibitor M04690 was developed as a potent inhibitor of the Wnt pathway and shows potential as a disease-modifying OA drug (DMOAD) [46]. Interestingly, to some extent, we noted that exosomal miR-92a-3p functions similarly to a Wnt inhibitor. In this study, exosomal miR-92a-3p inhibited WNT5A expression and enhanced aggrecan, COMP, and COL2A1 expression in chondrocytes.

To further explore the role of WNT5A in the regulation of cartilage degradation, we transfected PHCs with siWNT5A and found that COL2A1, COL9A1, aggrecan, and COMP mRNA expression was significantly upregulated, as was SOX9, aggrecan, and COL2A1 protein expression. This suggests that WNT5A may play an important role in cartilage degradation. Luciferase reporter assays showed that miR-92a-3p bound the 3′-UTR of *WNT5A* mRNA and downregulated its expression at the post-transcriptional level. Overall, our data showed opposing expression patterns between *WNT5A* and miR-92a-3p, indicating that miR-92a-3p regulates *WNT5A* expression during chondrogenesis.

Furthermore, in vivo, we investigate the role of MSC-miR-92a-3p-Exos by a collagenase-induced OA mice model. We found and determined that MSC-miR-92a-3p-Exos inhibit the progression of early OA and prevented the severe damage to knee articular cartilage in the OA mice model.

Although this study demonstrated that exosomal miR-92a-3p is efficacious in cartilage development and degeneration, there are still some limitations to our study. First, the detailed signaling pathways of the dual functions of WNT5A in MSCs undergoing chondrogenesis have not yet been clarified. We plan to conduct a cell signaling study in human bone MSCs to further elaborate the dual regulatory role of WNT5A in chondrogenesis. Furthermore, further studies are needed to clarify the specific function of exosomal-miR-92a-3p in the destabilization of the medial meniscus (DMM) model of OA and osteochondral defect model. For example, studies in rabbits or rats, in which exosome functions could be explored in combination with scaffolds for the treatment of cartilage defects and OA would be useful.

## Conclusions

The present study demonstrated that exosomal miR-92a-3p is expressed before and after chondrogenic differentiation in MSCs and is differentially expressed in OA. We found that exosomal miR-92a-3p functions as a negative regulator by downregulating WNT5A in both chondrogenesis and OA pathogenesis. We suggest that controlling the expression of exosomal miR-92a-3p has potential as a novel approach for prevention and treatment of OA.

## Additional file

Additional file 1: MiRNA expression profiles in exosomes before and after chondrogenic differentiation of MSCs. The expression profiles of miRNAs in exosomes before and after chondrogenic differentiation were detected using a miRNA microarray. SAM statistical software was used to identify differentially expressed miRNAs between undifferentiated MSC-Exos and chondro-differentiated MSC-Exos. Among the miRNAs that were consistently differentially expressed in all three paired samples, 36 were upregulated (fold change > 2) and 105 were downregulated (fold change <− 2) in chondro-differentiated MSC-Exos compared to levels in MSC-Exos. (XLSX 69 kb)

## Abbreviations

3′-UTR: 3′-untranslated region
ADAMTS: A disintegrin and metalloproteinase with thrombospondin motifs
DMEM: Dulbecco’s modified Eagle’s medium
ECM: Extracellular matrix
hMSC: Human bone marrow-derived MSCs
IL: Interleukin
MSC-Exos: Exosomes secreted by mesenchymal stem cells
miRNA: microRNA
MMP: Matrix metalloproteinase
MSC miR-92a-3p-Exos: Exosomes secreted by miR-92a-3p-overexpressing mesenchymal stem cells
OA: Osteoarthritis
PHC: Primary human chondrocyte
qRT-PCR: quantitative real-time reverse transcription-polymerase chain reaction
TGB: Transforming growth factor
